# Pharmacological effects of fibroblast growth factor 21 are sex-specific in mice with the lethal yellow (A^y^) mutation

**DOI:** 10.18699/VJ20.40-o

**Published:** 2020-03

**Authors:** E.N. Makarova, T.V. Yakovleva, N.Yu. Balyibina, K.O. Baranov, E.I. Denisova, A.D. Dubinina, N.A. Feofanova, N.M. Bazhan

**Affiliations:** Institute of Cytology and Genetics of the Siberian Branch of the Russian Academy of Sciences, Novosibirsk, Russia; Institute of Cytology and Genetics of the Siberian Branch of the Russian Academy of Sciences, Novosibirsk, Russia; Novosibirsk State University, Novosibirsk, Russia; The Institute of Molecular and Cellular Biology of the Siberian Branch of the Russian Academy of Sciences, Novosibirsk, Russia; Institute of Cytology and Genetics of the Siberian Branch of the Russian Academy of Sciences, Novosibirsk, Russia; Institute of Cytology and Genetics of the Siberian Branch of the Russian Academy of Sciences, Novosibirsk, Russia; Research Institute of Fundamental and Clinical Immunology, Novosibirsk, Russia; Institute of Cytology and Genetics of the Siberian Branch of the Russian Academy of Sciences, Novosibirsk, Russia Novosibirsk State University, Novosibirsk, Russia

**Keywords:** FGF21, A^y^-mice, melanocortin obesity, sex differences, liver, hypothalamus, FGF21, мыши A^y^, меланокортиновое ожирение, половые различия, печень, гипоталамус

## Abstract

Hypothalamic melanocortin 4 receptors (MC4R) regulate energy balance. Mutations in the MC4R gene are the most common cause of monogenic obesity in humans. Fibroblast growth factor 21 (FGF21) is a promising antiobesity agent, but its effects on melanocortin obesity are unknown. Sex is an important biological variable that must be considered when conducting preclinical studies; however, in laboratory animal models, the pharmacological effects of FGF21 are well documented only for male mice. We aimed at investigating whether FGF21 affects metabolism in male and female mice with the lethal yellow (A^y^) mutation, which results in MC4R blockage and obesity development. Obese C57Bl-A^y^ male and female mice were administered subcutaneously for 10 days with vehicle or FGF21 (1 mg per 1 kg). Food intake (FI), body weight (BW), blood parameters, and gene expression in the liver, muscles, brown adipose tissue, subcutaneous and visceral white adipose tissues, and hypothalamus were measured. FGF21 action strongly depended on the sex of the animals. In the males, FGF21 decreased BW and insulin blood levels without affecting FI. In the females, FGF21 increased FI and liver weight, but did not affect BW. In control A^y^-mice, expression of genes involved in lipid and glucose metabolism (Ppargc1a, Cpt1, Pck1, G6p, Slc2a2) in the liver and genes involved in lipogenesis (Pparg, Lpl, Slc2a4) in visceral adipose tissue was higher in females than in males, and FGF21 administration inhibited the expression of these genes in females. FGF21 administration decreased hypothalamic POMC mRNA only in males. Thus, the pharmacological effect of FGF21 were significantly different in male and female A^y^-mice; unlike males, females were resistant to catabolic effects of FGF21.

## Abstract (ru)

Гипоталамические меланокортиновые рецепторы 4-го типа (МК4Р) принимают участие в поддержа- нии баланса энергии. Мутации в гене, кодирующем МК4Р, – наиболее распространенная причина монолокусного ожирения у людей. Фактор роста фибробластов 21 (FGF21) рассматривают в качестве перспективного кандидата для медикаментозного лечения ожирения, однако неизвестно, влияет ли он на меланокортиновое ожирение. Пол особей необходимо учитывать при проведении доклинических исследований, но у лабораторных животных фармакологические эффекты FGF21 изучали только на самцах. В настоящей работе исследовано влияние FGF21 на метаболизм у самцов и самок мышей с мутацией lethal yellow в локусе агути (A^y^), которая приводит к блокаде МК4Р в гипоталамусе и развитию ожирения. Самцам и самкам мышей A^y^ линии C57Bl с развитым ожирением вво- дили подкожно в течение 10 дней физиологический раствор или FGF21 (1 мг/кг). Измеряли потребление пищи (ПП), массу тела (МТ), показатели крови и экспрессию генов в печени, мышцах, бурой жировой ткани, подкожной и висцеральной белой жировой ткани и гипоталамусе. Эффекты FGF21 зависели от пола животных. У самцов FGF21 снижал МТ и уровень инсулина в крови и не влиял на ПП. У самок FGF21 увеличивал ПП и массу печени, но не влиял на МТ. У контрольных самок A^y^ экспрессия генов углеводно-жирового обмена (Ppargc1a, Cpt1, Pck1, G6p, Slc2a2) в печени и генов липогенеза (Pparg, Lpl, Slc2a4) в висцеральной жировой ткани была выше, чем у самцов, и введение FGF21 снижало экспрессию этих генов только у самок. Введение FGF21 уменьшало уровень мРНК ПОМК в гипоталамусе только у самцов. Полученные результаты демонстрируют, что фармакологический эффект FGF21 значительно различается у самцов и самок мышей с мутацией A^y^: в отличие от самцов, самки проявляют устойчивость к катаболическому действию FGF21.

## Introduction

Obesity is a serious problem in modern society, this being the reason why various methods of combating obesity (medicinal, non-medicinal, preventive, etc.) are under intensive investigation. Hypothalamus plays a critical role in coordination of energy homeostasis, and mutations in various hypothalamic genes responsible for controlling appetite and metabolism lead to obesity (Singh et al., 2017). Melanocortin (MC) obesity, caused by mutations in the melanocortin system of the brain, is the most common genetic form of obesity in humans (Farooqi et al., 2003; Girardet, Butler, 2014). Melanocortin system regulates energy intake and expenditure. Activation of type 4 melanocortin receptors (MC4R) in the hypothalamic neurons reduces food consumption and increases energy expenditure, while their blockade or loss (knockout) is associated with hyperphagia, gradual development of obesity, and insulin resistance (Tao, 2010). In humans, the loss of MC4R functions causes severe obesity (Farooqi et al., 2003), but intensive search for therapeutic options of MC obesity correction has yet not identified an efficient drug (Fani et al., 2014).

Fibroblast Growth Factor 21 (FGF21) is assumed to be one of the most promising candidates for obesity treatment, because administration of FGF21 or its analogs was shown to reduce body weight in laboratory rodents, monkeys, and humans (Jackson et al., 2015). In rodents, it is efficient against both diet-induced and genetic forms of obesity (leptin ob/ob or its receptor db/db deficiency) (Kharitonenkov et al., 2005; Coskun et al., 2008). FGF21 is an atypical member of the fibroblast growth factor family; it possesses a hormone-like activity and is involved in maintaining energy homeostasis, regulation of carbohydrate and lipid metabolism, and adaptation to various stresses, including metabolic, such as nutritional deficiencies and calorie overload (Xie, Leung, 2017). FGF21 induces weight loss through its effects on the central nervous system (Lan et al., 2017). It is not known whether melanocortin system is involved in signal transmission from FGF21 to the CNS. If melanocortin signaling pathways are involved in the central action of FGF21, loss of function of the melanocortin system could reduce or eliminate the beneficial effect of FGF21 on metabolism and body weight. However, the pharmacological effects of FGF21 have not been studied in melanocortin obesity models.

Most animal studies of physiological and pharmacological effects of FGF21 have been made on males (Kharitonenkov et al., 2005; Coskun et al., 2008; Xu et al., 2009; Camporez et al., 2013; Markan et al., 2014). However, sex steroids have such a significant effect on the regulation of metabolic processes that National Institutes of Health (NIH) recognized sex as an important biological variable that must be considered when conducting preclinical studies (Mauvais-Jarvis et al., 2017; Clayton, 2018). In a few studies performed on rats and mice of both sexes, sex differences in the expression of FGF21 in liver (Lee et al., 2016; Chukijrungroat et al., 2017) and other tissues (Gasparin et al., 2018) were observed, exhibiting differential manifestation in obesity and starvation (Bazhan et al., 2019). These data suggest that the physiological and pharmacological effects of FGF21 may vary in individuals of different sexes.

The objective of this study was to investigate the pharmacological effects of FGF21 in male and female mice with melanocortin obesity. As a model of melanocortin obesity, we used mice with the lethal yellow mutation at the agouti locus (A^y^). In mice, A^y^ mutation causes ectopic overexpression of the agouti gene (Bultman et al., 1992). A^y^-mice have yellow coat color and develop obesity and non-insulin-dependent diabetes with age (Wolff et al., 1999), due to ectopic expression of agouti gene in the hypothalamus, which evokes chronic blockage of MC4Rs by the agouti protein (Michaud et al., 1997).

We found that therapeutic effects of FGF21 in A^y^-mice strongly depended on the sex. In male A^y^-mice, the blockage of MC4Rs did not prevent anti-obesity effect of FGF21, and its administration resulted in weight loss and decreased blood insulin levels. In females, FGF21 administration increased food intake without reducing body weight and glucose and insulin concentrations in blood, but inhibited the expression of genes related to glucose and lipid turnover in liver and increased liver weight. Thus, female A^y^-mice were resistant to anti-obesity effects of FGF21.

## Materials and methods

Ethical approval. All experiments were performed according to the Guide for the Care and Use of Laboratory Animals (1996) and the Russian National Instructions for the Care and Use of Laboratory Animals. The protocols were approved by the Independent Ethics Committee of the Institute of Cytology and Genetics (Siberian Branch of the Russian Academy of Sciences).

Animals. C57Bl and C57Bl-A^y^ mice were bred in the vivarium of the Institute of Cytology and Genetics in reciprocal crosses. The mice were separated from their mothers at the age of 4 weeks and housed in groups of 5–6 per cage. At the age of 30 weeks, each mouse was placed into a separate cage and housed individually until the beginning of the experiment. The mice were housed under a 12/12-h light-dark regime (light from 07:30 to 19:30) at an ambient temperature of 22–24 °C. The mice were provided ad libitum access to commercial mouse chow (Assortiment Agro, Turakovo Village, Moscow region, Russia) and water.

FGF21 (1 mg per 1 kg) or PBS were administered subcutaneously at the end of the light period (17:00–17:30) for 10 days. We have chosen this dose based on literature data. T. Coskun et al. (2008) showed that daily FGF21administration at this dose reduced body weight and blood glucose concentrations in male mice. To reveal the effect of FGF21 on glycaemia, fasted blood glucose was measured before and during the experiment. The mice were fasted overnight for two days before the first injection and at the seventh day of the experiment (after seven injections of FGF21 or PBS), and blood glucose was measured at the end of fasting. Glucose concentrations were measured using a Lifescan One Touch Basic Plus glucometer. Body weight and food intake were measured daily for 6 days prior to fasting and within 24 h of refeeding after fasting.

On the last day, the animals were sacrificed by decapitation (an hour after the injection), and samples of trunk blood were collected; liver, brown adipose tissue (BAT), and subcutaneous and abdominal white adipose tissues (WAT) were weighed, and the tissues were collected and snap-frozen in liquid nitrogen to evaluate gene expression. Seven male and six female mice received PBS (control); six male and five female mice received FGF21.

Plasma assays. Concentrations of insulin, leptin, and adiponectin were measured, respectively, using Rat/Mouse Insulin ELISA Kit, Mouse Leptin ELISA Kit (EMD Millipore, St. Charles, Missouri, USA), and Mouse Adiponectin ELISA Kit (EMD Millipore, Billerica, MA, USA). Concentrations of glucose, triglycerides and cholesterol were measured colorimetrically using, respectively, Fluitest GLU, Fluitest TG, and Fluitest CHOL (Analyticon® Biotechnologies AG Am Mühlenberg 10, 35104 Lichtenfels, Germany). Concentrations of free fatty acids were measured using NEFA FS DiaSys kits (Diagnostic Systems GmbH, Holzheim, Germany).

Expression and purification of mouse FGF21. Mouse FGF21 coding sequence (aa 29 to 210) was optimized for Escherichia coli expression and synthesized by Genewiz (South Plainfield, NJ, USA). This DNA sequence was subcloned into the expression vector pE-SUMOpro (LifeSensors Inc., USA). This construct was used for induction of fusion 6xHis-SUMO-fgf21 protein in E. coli BL21 (DE3) cells. The purified 6xHis-SUMO-fgf21 was cleaved using SUMO protease 1 and loaded onto a column with Ni-NTA resin. The FGF21 protein (aa 29 to 210) was in the flow-through fractions. Size exclusion chromatography on a Superdex 200 10/300 GL column was used as a final purification step. The absence of bacterial endotoxins in FGF21 protein sample was confirmed by LAL-test (< 0.2 U/μg protein).

Relative quantitation real-time PCR. Total RNA was isolated from tissue samples using ExtractRNA kit (Evrogen, Moscow, Russia) according to the manufacturer’s instructions. First-strand cDNA was synthesized using Moloney murine leukemia virus (MMLV) reverse transcriptase (Evrogen) and oligo(dT) as a primer. TaqMan gene expression assays (Applied Biosystems) listed in Table 1 were used for relative quantitation real-time PCR with β-actin as an endogenous control according to manufacturer’s manual. Sequence amplification and fluorescence detection were performed on an Applied Biosystems ViiA 7 Real-Time PCR System. Relative quantification was performed by the comparative threshold cycle (ΔΔCT) method.

**Table 1.**
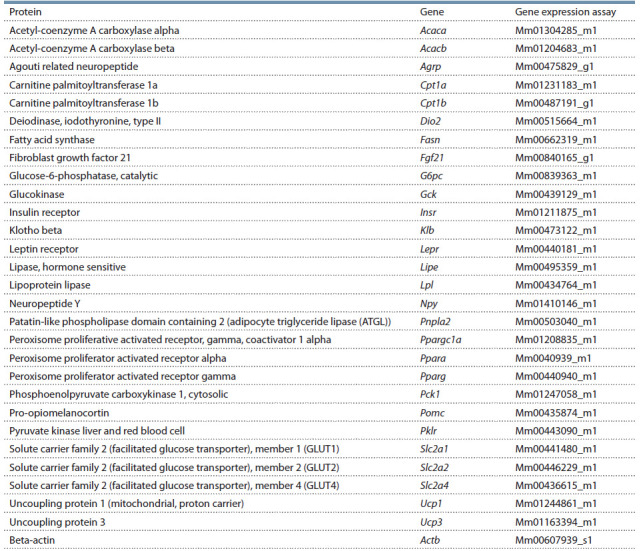
Gene expression assays used for relative quantitation real-time PCR

Statistical analysis. Each result is presented as an arithmetic mean ± SE for a sample size (i. e., number of mice) indicated. Three-way ANOVA with factors “sex” (male, female), “experimental group” (PBS, FGF21 administration), and “day of experiment” (1–6) was used to analyze FGF21 effects on food intake and body weight; with factors “sex”, “experimental group”, and “eating” (daily FI before and after fasting), to analyze FGF21 effect on FI after fasting. Two-way ANOVA with factors “sex” and “experimental group” was used to analyze FGF21 effects on blood parameters and gene expression with multiple comparisons using the post hoc Duncan test. Significance was determined as p ≤ 0.05. The STATISTICA 6 software package (StatSoft) was used for analysis.

## Results

Biochemical characteristics of blood and plasma. No differences between male and female A^y^-mice were observed in concentrations of blood glucose, either before or during the experiment. Only the plasma level of adiponectin was higher in females than in males. FGF21 administration significantly decreased plasma insulin specifically in male mice. Both male and female mice tended to respond to FGF21 administration by decreased plasma levels of free fatty acids (FFA) (Table 2).

**Table 2.**
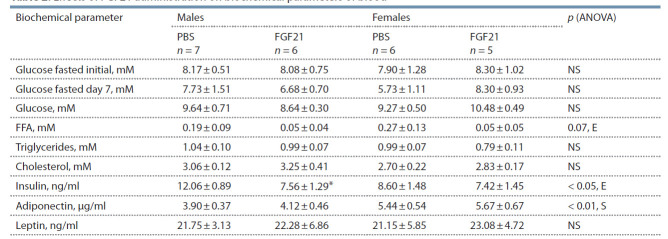
Effects of FGF21 administration on biochemical parameters of blood Male and female A^y^-mice were administered with FGF21 (1 mg per 1 kg) or PBS for 10 days, and blood samples to measure plasma parameters were collected 1 h after the last injection. Fasted glucose was measured in blood before (initial) and on day 7 of the experiment using a glucometer. *p < 0.05, males, FGF21 vs. PBS, post hoc Duncan test. NS – non-significant; S – “sex”; E – “experimental group”.

**Fig. 1.**
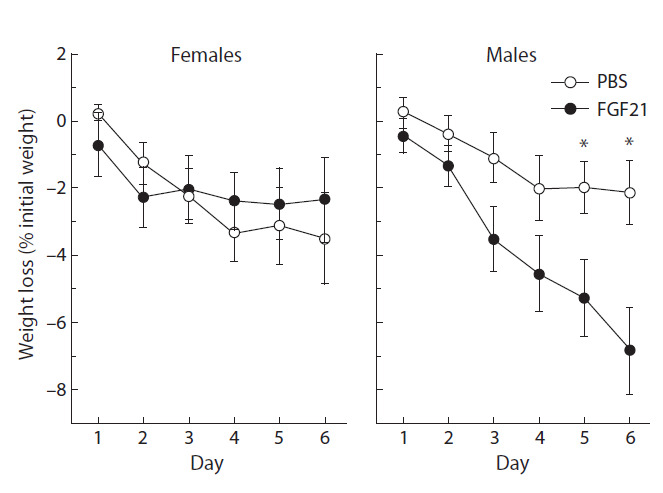
Effect of FGF21 administration on BW in male and female A^y^-mice. Weight loss was calculated as the difference between the weight on the day of injection and the initial weight, related to the initial weight (%). *p < 0.05, post hoc Duncan test.

Body weight (BW), weights of fat and liver, and food intake (FI). FGF21 administration exerted differential effects on BW in male and female mice ( p < 0.001, F_1.118_ ± 12.2, “sex” × “experimental group”, three-way ANOVA, Fig. 1). In males, FGF21 contributed to weight loss, and significant differences in BW between male mice treated with PBS and FGF21 were observed from day 5 of the experiment (see Fig. 1). In females, FGF21 administration did not affect BW.

**Fig. 2.**
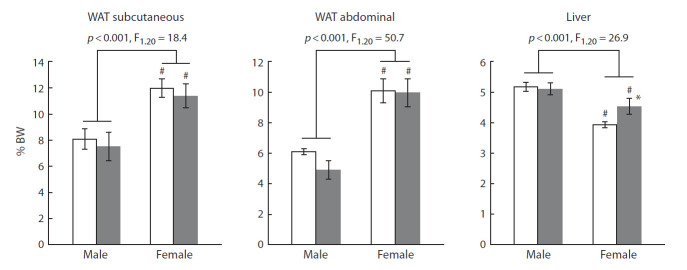
FGF21 influence on weights of fat tissues and liver in male and female A^y^-mice. Organ weights were calculated as percentages of BW. Sex dependence (two-way ANOVA) is indicated in the graph. #p < 0.05 for males vs. females; *p < 0.05 for FGF21 vs. PBS in females, post hoc Duncan test.

The weights of both subcutaneous and abdominal WAT were higher in female mice, whereas no such differences could be detected in the case of BAT, FGF21 administration did not affect fat weights in either males or females (Fig. 2). Liver weight was lower in females, and FGF21 administration increased this parameter in females, without affecting it in males (see Fig. 2).

**Fig. 3.**
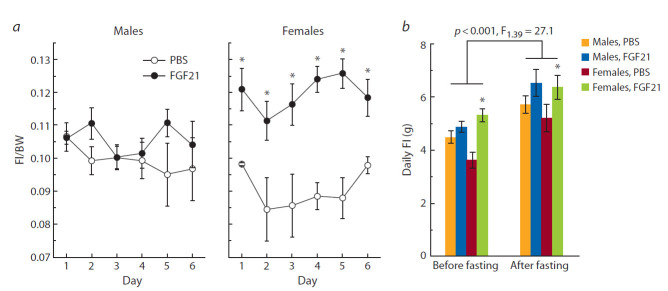
FGF21 influence on FI ad libitum (a) and after fasting (b) in male and female A^y^-mice. a – FI/BW ratio; b – mice were fasted overnight after seven injections of FGF21 or PBS. FI “before fasting” was calculated as an arithmetic mean of six daily FIs measured for 6 days prior to fasting. FI “after fasting” represents the amount of food consumed during 24 h of refeeding. The effect of fasting on FI (three-way ANOVA) is indicated in the graph. *p ≤ 0.05 for FGF21 vs. PBS in females, post hoc Duncan test.

The effect of FGF21 on FI was also sex-dependent ( p < 0.0001, F^1.113^ ± 20.7, “sex” × “experimental group”, three-way ANOVA, Fig. 3, a). FGF21 administration did not affect FI in males, but significantly increased it in females from the first day of the experiment (see Fig. 3, a).

We also assessed the effect of FGF21 on FI during the 24-h period of refeeding after overnight fasting. Fasting taken alone stimulated FI, and FGF21 administration further increased it during refeeding in both males and females ( p < 0.001, F^1.39^ ± 14.3, “experimental group”, three-way ANOVA, see Fig. 3, b).

**Fig. 4.**
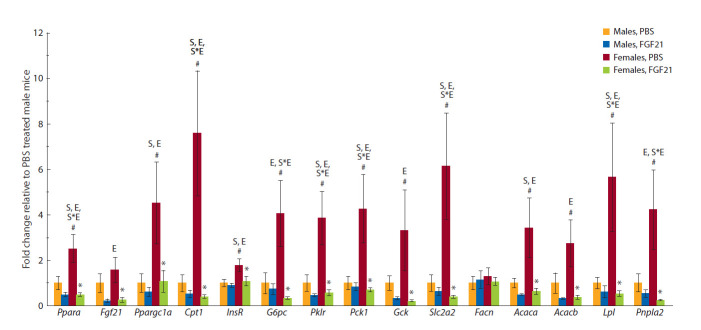
FGF21 influence on FI ad libitum (a) and after fasting (b) in male and female A^y^-mice. Effects of factors “S” (sex) or “E” (experimental group), or interactions thereof (“S*E”) at the level of significance p < 0.05 (two-way ANOVA) are indicated in the graph; #p < 0.05 for males vs. females; *p < 0.05 for FGF21 vs. PBS in females; post hoc Duncan test.

Gene expression. In mice that received PBS, liver expression of most of the genes studied was sex-dependent (Fig. 4).

**Fig. 5.**
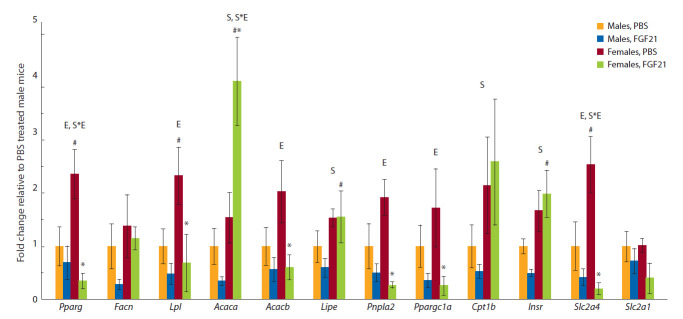
FGF21 influence on FI ad libitum (a) and after fasting (b) in male and female A^y^-mice. Effects of factors “S” (sex) or “E” (experimental group), or interactions thereof (“S*E”) at the level of significance p < 0.05 (two-way ANOVA) are indicated in the graph. #p < 0.05 for males vs. females; *p < 0.05 for FGF21 vs. PBS in females, post hoc Duncan test.

Higher levels of mRNA of genes involved in beta-oxidation (Ppara, Pgc1, Cpt1), glucose metabolism (Insr, Slc2a2), glycolysis (G6pc, Pklr), and gluconeogenesis (Gck, Pck1), as well as lipolysis (Pnpla2) and lipogenesis (Lpl, Acaca, Acacb) were observed in females, as compared to males. FGF21 administration inhibited liver expression of the genes, and this inhibition was more pronounced in females than in males. Thus, FGF21 administration effectively eliminated sex differences in liver expression of the genes, observed in the control (see Fig. 4). Liver expression of Fgf21, exhibiting no differences between males and females, was reduced in response to exogenous FGF21. Sex differences in gene expression were also observed in abdominal fat. In mice treated with PBS, mRNA levels of genes encoding PPARg, LPL, and GLUT4 were higher in females than in males (Fig. 5). FGF21 administration reduced the expression of these genes in females to a greater extent than in males, thereby eliminating the sex-dependent differences observed in the control (see Fig. 5).

For the majority of the genes studied, the inhibitory effects of FGF21 administration on the expression were more pronounced in females. This was confirmed for genes involved in lipogenesis (Pparg, Lpl, Acacb), lipolysis (Pnpla2), and transcription coactivation (Ppargc1a). In a single case of Acaca gene, the pattern was reversed: FGF21 administration decreased the level of mRNA in males while increasing it in females. The expression of Insr and Lipe was higher in females, and FGF21 administration did not affect their mRNA levels (see Fig. 5).

In subcutaneous fat of the controls, there were no sexdependent expression differences for any of the genes studied. FGF21 administration affected only Cpt1b, decreasing its expression both in males and females.

In brown fat of the controls, there were no sex-dependent expression differences for any of the genes studied, and FGF21 administration did not affect the expression.

In muscle tissue of the controls, there were no sex-dependent expression differences for any of the genes studied. FGF21 did not affect the expression significantly, although the level of Cpt1b mRNA tended to increase after FGF21 administration ( p ± 0.06, two-way ANOVA).

Mice treated with PBS exhibited no differences between males and females in the hypothalamic mRNA levels of the genes studied. After FGF21 administration, the expression of Klb was decreased in mice of both sexes ( p < 0.05, F^1.20^ ± 6.2), whereas that of Pomc was decreased in males only ( p < 0.05, Student’s t-test, 1.0 ± 0.3, n ± 6, control males vs. 0.29 ± 0.10, n ± 6, FGF21 treated males).

## Discussion

In the present study, we assessed the pharmacological effects of FGF21 in male and female mice with A^y^ mutation, which evokes MC4R blockage. We found that these effects were strongly dependent on the sex of the animals.

FGF21 administered to animals with diabetes and obesity decreases their BW and blood glucose/insulin levels, improves blood lipid profile, and increases insulin sensitivity (Bon- Durant, Potthoff, 2018). In our study, responses to FGF21 administration in male A^y^-mice were largely consistent with its expected impact: their BWs and blood insulin decreased, blood levels of FFA tended to decrease, and blood glucose and adiponectin were not affected. Our observation that FGF21 decreased blood insulin without affecting glucose, which retained normal levels, suggests that insulin sensitivity in male A^y^-mice was possibly improved. Metabolic effect of FGF21 administration was not associated with changes in blood adiponectin, since adiponectin is not required for the chronic effects of FGF21 to reduce body weight or its effects on glucose homeostasis (BonDurant et al., 2017).

Although we did not monitor energy expenditure, it was most likely increased, since weight loss in male mice took place in the absence of changes in FI. The ability of FGF21 to induce weight loss without affecting FI was reported previously for male mice with diet-induced and genetic obesity (db/db, ob/ob) (Kharitonenkov et al., 2005; Xu et al., 2009; Camporez et al., 2013). This effect was arguably due to increased energy expenditure caused by intense locomotor activity (Xu et al., 2009) and elevated metabolic rate (Coskun et al., 2008; Xu et al., 2009). FGF21-induced growth of metabolic rates is associated with augmentation of fatty acid oxidation in liver and adipose tissue (BAT and WAT), caused in turn by increased expression of genes encoding CPT-1 (in liver only), PGC-1 (in liver and adipose tissue), and UCP-1 (in liver and adipose tissue) (Camporez et al., 2013). No such changes in gene expression have been observed in this study; on the contrary, FGF21 administration to males reduced the expression of Ppargc1a and Cpt1 in liver and Ppargc1a in abdominal WAT ( p < 0.05, two-way ANOVA).

Our results are consistent with those reported by (Coskun et al., 2008), demonstrating that weight loss and improved glucose metabolism in FGF21-treated C57Bl males were accompanied by a decrease in liver expression of Cpt1 (with no changes of Ppargc1a expression). However, T. Coskun et al. (2008) found increased expression of genes related to (1) thermogenesis (Ucp1) in WAT and BAT, (2) lipolysis (Lipe, Pnpla2) in WAT, and (3) lipogenesis (Acaca, Acacb) in WAT, suggesting that energy expenditure is achieved via thermogenesis and induction of a state of increased futile cycling. We have not observed induction of Ucp1 expression either in BAT or WAT; likewise, there have been no increase in the expression of lipolysis/lipogenesis genes in liver or adipose tissue. Thus, our experimental data provide no indication of futile cycling activation. FGF21-induced activation of energy expenditure involves, in addition to central mechanisms, the sympathetic nervous system (Owen et al., 2014; Lan et al., 2017). Of note, the latter also mediates regulation of energy expenditure by the melanocortin system (Rossi et al., 2011; Berglund et al., 2014). Therefore, FGF21 may act, at least in certain cases, via melanocortin signaling pathways. If so, the blockade of MC4R in A^y^-mice can interfere with the activating effect of FGF21 on the expression of the genes we studied. Weight loss in male A^y^-mice is indicative of yet other mechanisms whereby energy expenditure may be increased, which remain to be explored.

In females with A^y^ mutation, the effect of FGF21 differed dramatically from that observed in males. In females unlike males, FGF21 administration did not induce weight loss, but increased liver weight. A^y^-mice are hyperphagic (Wolff et al., 1999), and FGF21 exacerbated hyperphagia specifically in females. It is not known whether this difference is due to A^y^ mutation or, rather, we are dealing with a general sex-linked discrepancy in responses of female and male mice to FGF21 treatment (the pharmacological effects of FGF21 have thus far not been studied in female mice).

Resistance of female A^y^-mice to catabolic effects of FGF21 can be explained by increase of food intake. FGF21 influence on FI in female rodent was not studied, but in males, FGF21 was shown previously to increase FI in rats (Recinella et al., 2017), mice with diet-induced obesity (Coskun et al., 2008), and to increase protein intake while reducing carbohydrate intake in normal mice, and the later effect was mediated via CNS (Larson et al., 2019). However, FGF21-dependent signaling pathways that regulate eating behavior have not been identified. The melanocortin system is involved in the regulation of the response to protein deficiency, and FGF21 may act as the sensor triggering that response. In our experiment, male mice demonstrated no increase in FI, although FGF21 administration decreased hypothalamic expression of Pomc. POMC is a precursor of the anorexigenic neuropeptide MSH, and a decrease in Pomc expression (limiting MC4R activation by MSH) is associated with increased FI. A^y^ mutation leads to MC4R blockade, which disrupts MSH regulation of FI and may be the reason why males did not respond to FGF21 by increased FI.

In females, FGF21 administration caused a considerable increase in FI without affecting the expression of the melanocortin system genes. In A^y^-females, FGF21 influence on FI may be mediated via estradiol-sensitive mechanism that is not altered by impaired melanocortin signaling (Morton et al., 2004). It is possible that the orexigenic effects of FGF21 in males and females involve distinct neuronal signaling pathways.

According to the data obtained in the control groups, a different metabolic response to exogenous FGF21 in males and females was induced at the background of significant sex differences in the mass of the liver and adipose tissue and in the expression of the liver and fat genes. Sex differences in gene expression in adipose tissue differed depending on localization; in our experiment, we found them only in abdominal adipose tissue. These data are consistent with the results obtained when assessing gene expression in adipose tissue in mice: the number of differentially expressed genes in males and females was significantly higher in abdominal than in subcutaneous fat (Grove et al., 2010). Increased weight of abdominal fat in control A^y^-females, compared to males, was associated with increased expression of genes upregulating lipogenesis in adipose tissue: Ppar, Lpl, Insr and Slc2a4. FGF21 administration eliminated differences in the expression of these genes, however, it did not lead to significant changes in the mass of adipose tissue. It is possible that more prolonged FGF21 administration is required to detect changes at the level of adipose tissue weight.

The most striking sex differences in gene expression in the control groups were observed in liver. In controls, the expression of most genes studied (except for Fgf21 and Fasn) was higher in females than in males. These results are consistent with transcriptome analysis data demonstrating differential expression rates for 72 % active liver genes in male and female mice (Yang et al., 2006). Sex differences in expression of genes related to lipid metabolism were found in the liver of mice and rats on high-calorie diet: compared to males, females had enhanced mRNA levels of Acc1, Pparg, Cpt1 and other genes in mice (Gasparin et al., 2018), and enhanced protein levels of ACC, PPARs and FAS in rats (Chukijrungroat et al., 2017). As shown by S. Della Torre et al. (2017), liver is the major target for estrogens, and estrogen receptor alpha (ERα) has a direct effect on the regulation of the hepatic genes relevant for energy metabolism.

Increased expression of genes involved in fatty acid β-oxidation (Ppara, Ppargc1a, Cpt1), glycolysis (G6p, Pklr), gluconeogenesis (Pck1, Gck), glucose transport (Slc2a2), lipogenesis and lipolysis (Acaca, Acacb, Lpl, Pnpla2) possibly indicates that the rate of glucose and fat metabolism is higher in the liver of A^y^-females than A^y^-males. FGF21 administration reduced liver expression of all the genes mentioned, thereby eliminating entirely any sex-dependent differences, and this effect was associated with the increase of female liver weight. Enlarged liver under the action of FGF21 in female A^y^-mice may indicate the development of steatosis. Simultaneous decrease in catabolic (lipolysis, glycolysis) and anabolic (lipogenesis, gluconeogenesis) processes can suggest decreased metabolism in the liver of A^y^-females. Decreased liver metabolism in females could reduce liver energy expenditure and contribute, together with increased food intake, to liver weight gain. Additional morphological and biochemical studies are needed to find out whether FGF21 initiates (or promotes) steatosis in A^y^-female mice.

Taken together, our findings indicate that therapeutic effects of FGF21 in mice with disrupted melanocortin signaling are strongly sex-dependent. In male A^y^-mice, the blockage of MC4Rs does not prevent the anti-obesity effect of FGF21: its administration results in weight loss and blood insulin decrease. However, obese A^y^-females exerted resistance to catabolic and antidiabetic effects of FGF21. In females, exogenous FGF21 stimulates FI without reducing BW and blood glucose/insulin, inhibits liver expression of genes related to glucose and lipid turnover, and increases liver weight. The contribution of estrogens and the melanocortin system to those effects remains to be elucidated.

## Заключение

The pharmacological effect of FGF21 may depend on the animal sex and etiology of obesity. Although an immediate translation to humans of findings obtained in experiments with mice is not possible, our results suggest that detailed preclinical studies of the pharmacological effects of FGF21 are required, taking into account the sex of individuals under study and the genesis of obesity.

## Conflict of interest

The authors declare no conflict of interest.
